# Computational optimization problems in social interaction and empathic social emotion

**DOI:** 10.1186/1471-2202-15-S1-P35

**Published:** 2014-07-21

**Authors:** Nicoladie D Tam

**Affiliations:** 1 Department of Biological Sciences, University of North Texas, Denton, TX 76203, USA

## 

Social interaction is a computational problem that requires optimization among multiple agents in social group, such as optimization in human interactions and swamp robot interactions. A social group is a group of autonomous agents (humans, animals or any autonomous robots) that interact with each other to form an inter-dependent group as a system. The dynamics of interaction can vary from cooperation, collaboration, commissural and competition, which can be beneficial or detrimental to the group and/or individuals. Toward the goals of understanding the dynamics of such a socially interactive group, the computational problem can be reduced to an optimization problem of gains and losses relative to the individuals as well as relative to the group. In a cooperative social environment, the optimization is to maximize the gains for both the individuals and the group. In a competitive social environment, the optimization is to maximize the gains for the individual self while minimizing the gains for other individuals.

In this study, I have derived a computational social interaction model that incorporates the empathic social emotion as an implicit optimization variable to extend the concept of “self” to include “others” (as a part of the “extended self”) to achieve cooperative social interactions even in a competitive environment. The model uses the optimizing computation based on survival principles, in which the individual self will attempt to maximize gains while minimize losses for self. Furthermore, the gains for self take priority over the gains for others in survival principles for self-preservation. Yet, when the goal of the optimization process is to maximize gains for “self” over “others,” it will result in the competitive social interaction where the gains are maximized for “self” (as in selfishness) while the losses are maximized for “others” (as in combativeness). Based on this optimization principle, cooperative social interactions cannot be achieved when self-interests take priority/precedence over others-interests. This often leads to destruction of others in social competition, rather than mutual preservation in social cooperation. In order to achieve cooperative behavior while not violating the optimization principle of self-preservation, I derived an “empathy model” as a social emotion in which the individual self is extended to include other agents (other individuals) as a part of the “extended self”. When other individuals are included as a part of the extended self, then optimization can be maximizing gains for both self and others simultaneously, without compromising the survival principles that call for maximizing gains for self only while maximizing losses for others (because the extended self now includes both self and others). This extended entity to incorporate others as a part of the extended self provides the basis for the development of empathy and empathic emotions, which is the ability to feel for others. It also serves as the basis for compassion, which is an empathic emotion that does not just feel for others, but also motivates to minimize the losses for others (rather than maximize the losses for them in the process of maximizing gain for self). The above social emotion model is an extension of the computational models of EMOTION-I [1] and EMOTION-II [2] that are used to derive self-emotions (emotions feedback computed relative to self but not others) based on survival principles. This current model extends the previous models to include other individuals as a part of self in the derivation of social emotions, in addition to the mathematical derivations of self-emotions described earlier [3-6]. These self- and social-emotion models form the basis for solving the optimization problems for maximizing gains for self without necessarily creating conflicts in maximizing losses for others in a cooperative social environment. This extended-self model of empathy and compassion can now be used to explain the social behaviors in maternal love (mother-child interaction) and romantic love (pair-bonding interaction) using an optimization model without requiring other psychological principles or anthropological rationales for the evolution of empathic love and cooperative behaviors in social interactions.
